# Trinocular Vision-Driven Robotic Fertilization: Enhanced YOLOv8n for Precision Mulberry Growth Synchronization

**DOI:** 10.3390/s25092691

**Published:** 2025-04-24

**Authors:** Ma Ming, Osama Elsherbiny, Jianmin Gao

**Affiliations:** School of Agricultural Engineering, Jiangsu University, Zhenjiang 212013, China; 2212216062@stmail.ujs.edu.cn (M.M.); osama_algazeery@mans.edu.eg (O.E.)

**Keywords:** trinocular vision, deep learning, mulberry cuttings, foliar fertilizer, upgraded YOLOv8n, parallel robotics

## Abstract

This study focused on addressing the issue of delayed root system development in mulberry trees during aerosol cultivation, which is attributed to the asynchronous growth of branches and buds. To tackle this challenge, we propose an intelligent foliar fertilizer spraying system based on deep learning. The system incorporates a parallel robotic arm spraying device and employs trinocular vision to capture image datasets of mulberry tree branches. After comparing YOLOv8n with other YOLO versions, we made several enhancements to the YOLOv8n model. These improvements included the introduction of the Asymptotic Feature Pyramid Network (AFPN), the optimization of feature extraction using the MSBlock module, the adoption of a dynamic ATSS label assignment strategy, and the replacement of the CIoU loss function with the Focal_XIoU loss function. Furthermore, an artificial neural network was utilized to calculate the coordinates of the robotic arm. The experimental results demonstrate that the enhanced YOLOv8n model achieved an average precision of 94.48%, representing a 6.05% improvement over the original model. Additionally, the prediction error for the robotic arm coordinates was maintained at ≤1.3%. This system effectively enables the precise location and directional fertilization of mulberry branches exhibiting lagging growth, thereby significantly promoting the synchronous development of mulberry seedlings.

## 1. Introduction

Atomization reduces the waste of water and nutrient solutions and effectively prevents the spread of fungal diseases [1,2]. In the process of atomization cultivation, the environmental factors in the space where the plant’s root zone is located can be artificially controlled [3,4,5]. Moreover, aeroponic crops grow more rapidly [6,7,8]. However, different buds grow at different rates, with some taking longer to break dormancy, leading to inconsistent development and delays in root formation. Therefore, it is necessary to identify and distinguish mulberry cuttings in an aeroponic system and spray foliar fertilizer to promote the growth of the slow-growing ones. Selectively spraying foliar fertilizer on slow-growing mulberry cuttings saves more fertilizer than spraying foliar fertilizer on all mulberry cuttings, reduces the operating time of the robotic arm, and lowers production costs. In large-scale mulberry aeroponics, manually identifying the growth status of mulberry cuttings is not only time-consuming but also incurs significant costs. With the development of science and technology, deep learning and machine vision technology are gradually being applied to the field of object recognition. Accurately identifying slow-growing individuals in crops using these technologies is of great significance for agricultural production, as it allows for the targeted spraying of nutrient solutions to promote growth [9,10,11]. Precise fertilization, achieved by accurately identifying slow-growing crops and spraying them, is crucial for the development of precision agriculture and intelligent agriculture [12,13,14,15].

Parallel robotic arms offer several advantages, including higher stiffness, precision, and load-bearing capacities. These features make them particularly well suited for complex agricultural environments [16,17,18,19]. Consequently, we selected a parallel robotic arm as the primary actuator. As demonstrated in Lu S et al. [20], the minimum-jerk trajectory planning approach further enhances parallel robotics. It ensures smooth and precise motion, reduces vibrations, and improves the overall performance and reliability.

In recent years, to tackle the challenges of intelligent recognition and detection in complex environments, researchers across the globe have increasingly turned to deep learning methods [21,22,23]. Xu et al. [24] enhanced YOLOv5 by integrating the Mish activation function, employing DIoU_Loss to accelerate bounding box regression, and incorporating the Squeeze Excitation module. These modifications resulted in a grading precision of 90.6% and a real-time processing speed of 59.63 FPS, significantly boosting both the precision and detection efficiency for apple grading tasks. Ji W et al. [25] improved the YOLOv5s model by adding ODConv and GSConv convolutions, along with a VoVGSCSP lightweight backbone. This allowed for simultaneous apple surface defect detection and fruit stalk identification, focusing on side information from multi-view images. Their model achieved 98.2% precision in defect detection, processing 30 fps. Ji W et al. [26] proposed a ShuffleNetv2-based apple object detection model, integrating an adaptive spatial feature fusion (ASFF) module into the PANet network. The model attained 96.76% average precision, 95.62% precision, 93.75% recall, and a 0.95 F1 score, with a detection speed of 65 FPS. Zhu W et al. [27] introduced the Transformer Encoder and CenterLoss in an improved model to establish an accurate and efficient disease recognition model. Liu S et al. [28] designed a tomato flower pollination feature recognition method based on the deep learning fully open flower recognition model and the binocular template matching three-dimensional information recognition method. Zhang Z et al. [29] constructed an all-weather lightweight tea crown shoot detection model (TS-YOLO) by replacing the feature extraction network of YOLOv4 and the standard convolution of the whole network with a lightweight neural network, MobilenetV3, and depth-separable convolution, among other improvements. The size of the improved model was 11.78 M, which was 18.30% of the size of YOLOv4, and the detection speed was improved by 11.68 FPS. Zhang F et al. [30] proposed the Feature Enhancement Network Block (FENB) based on the YOLOv4-Tiny model. They designed the FENB using the CSPNet structure with a hybrid attention mechanism and constructed a Feature Enhancement Network (FEN) on top of the FENB to enhance the feature extraction capability and improve the detection accuracy of YOLOv4-Tiny. In a study by Wu F et al. [31] considering an insufficient dataset of nighttime images and the problems of the poor detail restoration and color distortion of existing CycleGAN models, an enhanced CycleGAN method integrating style migration and small sample detection was proposed. By introducing the ResNeXtBlocks generator and optimizing the upsampling module and the hyperparameter strategy, the FID score was reduced by 29.7%, and the precision, recall, and other metrics were improved by 13.34–56.52% compared to the YOLOv7 detection framework.

Most existing recognition methods rely primarily on monocular and binocular image recognition techniques [32,33,34], with limited research on trinocular and multi-eye image recognition methods. Additionally, there is a scarcity of studies focusing on the identification of a need for fertilizer, the location of crops, and the targeted spraying of liquid fertilizer for both fast- and slow-growing crops under identical conditions. To enhance recognition precision, we conducted a comparative analysis of the detection capabilities of visual systems with varying numbers of cameras, specifically for mulberry tree cuttings. Our investigation indicated that the trinocular vision system effectively reduced occlusion between mulberry tree cuttings, thereby enhancing the detection accuracy and overall performance.

The YOLOv8 model appears to be highly mature in practical applications, with significant performance improvements observed in its enhanced versions. For instance, Hemamalini et al. [35] successfully increased the model’s average precision to 99.2% for plant thermal canopy detection by integrating the compact YOLOv8-C detection technology with the innovative Fast Segment Anything Model (FastSAM) method, thereby greatly enhancing the model’s overall performance. Similarly, Xu J et al. [36] built upon the YOLOv8 base model by incorporating the Large Separable Kernel Attention (LSKA) mechanism into SPPF and replacing YOLOv8’s Neck with an optimized Slimneck module to develop the SLPD-YOLOv8 model. This improved model achieved an accuracy of 94.8% in recognizing the number of stress cracks in corn seeds, significantly boosting the model’s detection capabilities.

In this study, the primary challenge addressed was the inefficient detection and control of robotic arms in complex agricultural environments, particularly for tasks such as foliar fertilizer spraying on mulberry branches. Traditional detection models and robotic control systems often struggle with occlusions, dense foliage, and the need for precise spatial data. To tackle these challenges, we developed an intelligent mulberry foliar fertilizer spraying system that leverages advanced detection and control methodologies. Specifically, we optimized the YOLOv8n model by introducing the Asymptotic Feature Pyramid Network (AFPN) in the Neck part, fusing the C2f module with MSBlock, replacing the CIoU loss function with XIoU, and integrating the DynamicATSS module. These enhancements significantly improved the detection ability of the YOLOv8n model. In addition, we introduced a multi-camera hybrid data fusion approach to capture spatial diversity, leveraging artificial neural networks (ANNs) to merge and analyze 3D positional data. This method effectively compensated for occlusions and improved the reconstruction precision in dense foliage. Thus, the main objectives of this study were (1) to develop an intelligent mulberry foliar fertilizer spraying system that supports the Internet of Things (IoT) and promotes the growth of slow-growing mulberry cuttings by accurately identifying them for foliar fertilizer spraying; (2) to evaluate different YOLO versions, including YOLOv8n and YOLOv10, to determine their effectiveness in complex agricultural environments; (3) to optimize the YOLOv8n model by introducing the AFPN in the Neck part, fusing the C2f module with MSBlock, replacing the CIoU loss function with XIoU, and integrating the DynamicATSS module, thereby significantly enhancing its detection ability; and (4) to create computational control frameworks for robotic manipulator systems using ANNs to improve the adaptability and precision of robotic arm control in dense foliage.

## 2. Materials and Methods

### 2.1. Aeroponic Cultivation of Mulberry Cuttings

**Figure 1:**
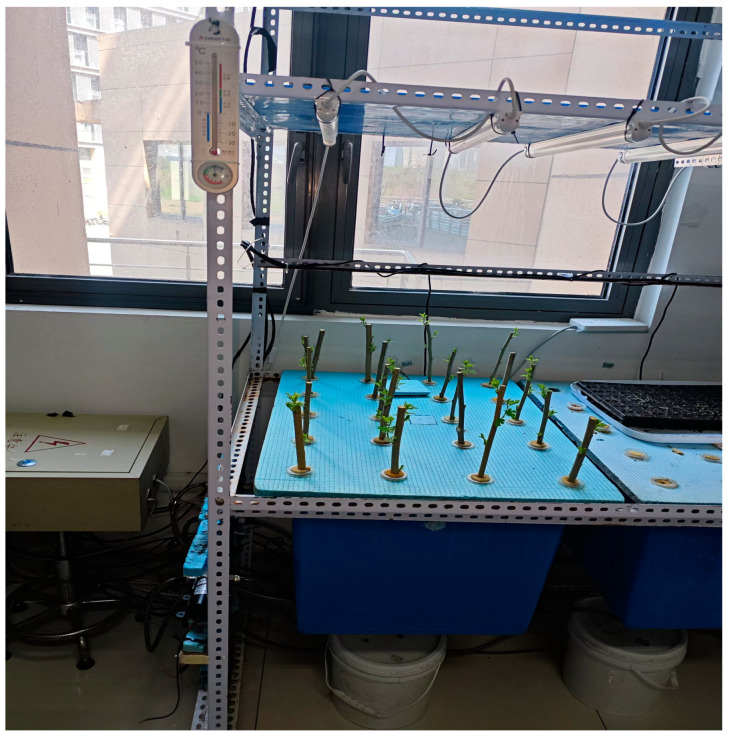
Schematic diagram of a fog incubator.

An experiment exploring the fogging cultivation of mulberry cuttings was conducted in the fogging laboratory at Jiangsu University. Healthy, pest-free cuttings with strong vitality and new shoots were selected. From these, 15–18 cm branches bearing 2–4 shoots were carefully cut. The mulberry cuttings were sterilized with a potassium permanganate solution for 30 min, and after sterilization, they were rinsed with water and dried. Then, the cuttings were soaked in a rooting solution for 2 h and transferred to a mist incubator. The fog box was an HDPE box with dimensions of 740 mm (length) × 535 mm (width) × 415 mm (height). We used a piece of foam board with dimensions of 730 mm (length) × 525 mm (width) × 10 mm (height) as a colonization plate to place on the fog box. Twenty holes with a diameter of 34 mm were drilled at intervals of 14 cm by 14 cm as planting holes, and planting baskets with an inner diameter of 32 mm and an outer diameter of 45 mm were placed inside the planting holes. A sponge with a hole in the center was placed inside a planting basket, a mulberry cutting was placed in the sponge, and the lower part of the mulberry cutting was suspended inside the fog cultivation box through the hole at the top of the planting basket. A schematic diagram of the fog incubator is shown in Figure 1. Four nozzles were mounted at the bottom of the fog cultivation tank, and the four nozzles were connected to a water pump (PLD-1206, 12 V, 3.5 A, 4 L/min) through a PU air hose. In the first week, fresh water (tap water) was employed in the fog culture system, and in the second week, the fresh water was replaced with the soilless nutrient solution (inorganic fertilizer solution) A and B from the Air Garden brand. This nutrient solution, mixed with fresh water at a ratio of 1:400 by volume, was used to spray the mulberry cuttings. The water pump (PLD-1206, 12 V, 3.5 A, 4 L/min) was made via a Raspberry Pi to spray the nutrient solution for 2 min every 2 h. After spraying the nutrient solution for 8–10 days, the shoots of the mulberry tree cuttings gradually began to grow new leaves. However, some branches showed delayed growth and required a longer period to develop. A solution of potassium dihydrogen phosphate with a mass fraction of 0.4% was prepared as a mulberry foliar fertilizer solution, and the mulberry cuttings were put into the developed mulberry foliar fertilizer intelligent spraying system. After the intelligent spraying system identified and located a slow-growing mulberry cutting, the robotic arm moved the 0.5 mm nozzle to 30 mm above the slow-growing mulberry cutting and then turned on the micro-peristaltic pump (5 V, 1 A, 1.5–3.2 L/min) to spray the foliar fertilizer solution for 20 s, controlled using a Python program.

### 2.2. Robotic Arm-Based Foliar Fertilizer Spraying for Mulberry Trees

#### 2.2.1. System Architecture

**Figure 2:**
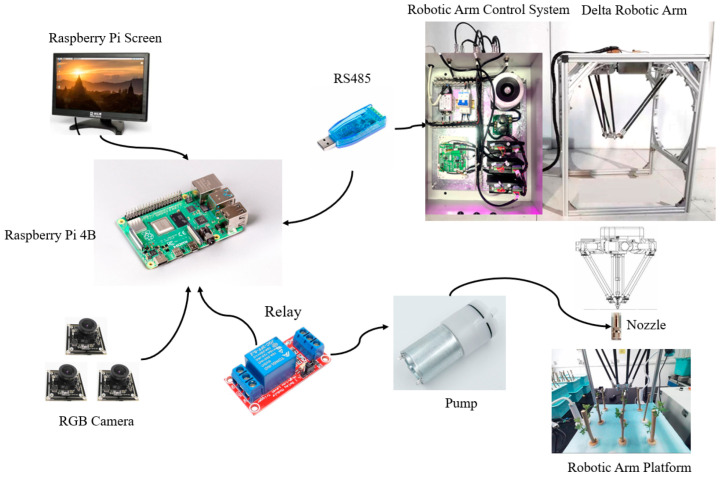
Schematic of foliar fertilizer spraying system for aeroponic mulberry trees using parallel robotic arm.

**Figure 3:**
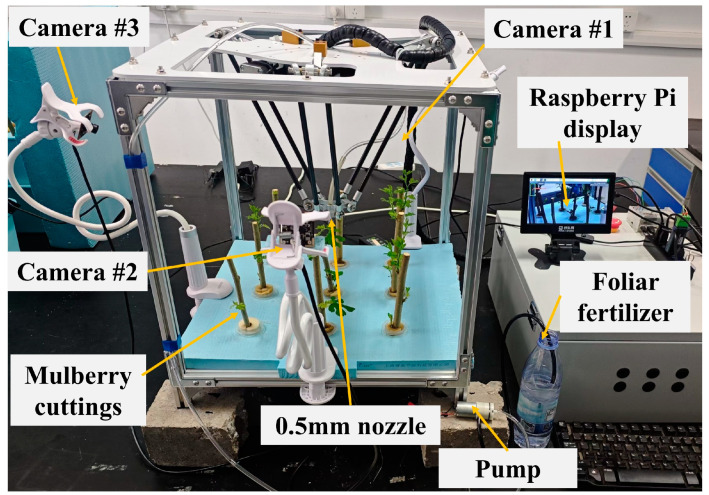
Physical diagram of the foliar fertilizer spraying system for aeroponic mulberry cultivation.

The foliar fertilizer spraying system for aeroponic mulberry trees, which incorporates a parallel robotic arm, is composed of two primary components: the parallel robotic arm and the control system. Figure 2 shows a schematic diagram of the foliar fertilizer spraying system for aeroponic mulberry trees equipped with a parallel robotic arm, and Figure 3 displays a physical diagram of the same system.

The parallel robotic arm consists of 6 manipulator connecting rods, 12 rings, 6 fisheye bearing tie rods, an end effector, 3 motor drive rods, and an external frame. The two ends of the connecting rods are connected to the rings, with the rings mounted on the fisheye bearings of the fisheye bearing tie rods. The ring at the upper end of the robotic arm’s connecting rod, along with the attached fisheye bearing tie rod, forms the joint of the robotic arm, which provides 3 degrees of freedom. One end of the motor drive rod is connected to the motor shaft to transmit power, while the other end is connected to the fisheye bearing pull rod, which is linked to the upper end of the mechanical arm connecting rod, driving the movement of the arm. The end effector is connected to the fisheye bearing pull rod attached to the lower-end ring of the arm’s connecting rod to carry out the robotic arm’s operational tasks.

**Figure 4:**
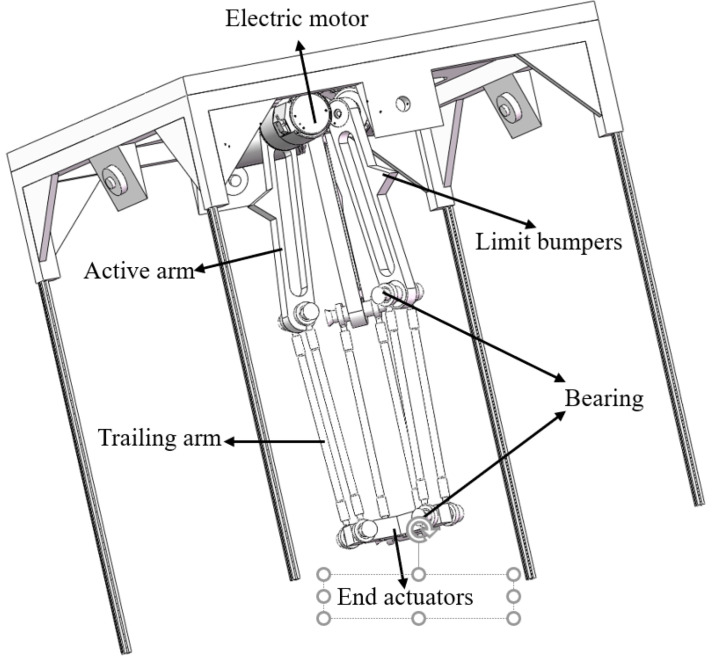
Three-dimensional schematic of a parallel robotic arm.

The parallel robotic arms are positioned within a cube frame with dimensions of 46 cm in length, width, and height. The origin of the robotic arm’s coordinate system lies along the same line as the center points of the upper and lower square frames. A three-dimensional model of the parallel robotic arm is shown in Figure 4. After the slow-growing mulberry cuttings are identified and located, the sprinkler on the end effector is made to move above the corresponding cuttings, and the micro-peristaltic pumps are activated to spray foliar fertilizer.

The parallel robotic arm in Figure 4 comprises several key components: The electric motor receives and converts control signals from the motor driver to drive the active arm movement. Limit bumpers trigger the limit switch through physical contact when the robot arm reaches its limit position, ensuring the safety control of the movement range. The active arm, powered by the drive device, executes core motion commands and drives follower parts to perform precise tasks like positioning, gripping, and handling. The trailing arm transmits the movement and force from the active arm, assisting in precise positioning and stabilizing the end effector while enhancing the overall structural rigidity and coordination. Bearings support moving parts, reduce friction, and ensure flexible, high-precision relative motion between chains, maintaining stability and coordinated movement. Finally, end actuators make direct contact with the object, performing specific tasks such as grasping, processing, and inspection.

#### 2.2.2. Control System Design Scheme

**Figure 5:**
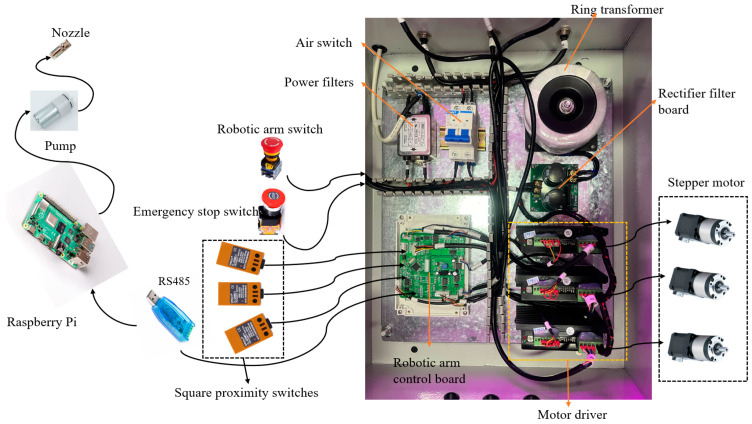
Foliar fertilizer spraying control system for mulberry trees with a parallel robotic arm.

The design of the foliar fertilizer spraying control system with a parallel robotic arm for aeroponic mulberry cultivation is shown in Figure 5. The control system consists of a Raspberry Pi, a robotic arm control board, a power plug, a robotic arm switch, an emergency stop switch, a power filter, an air switch, a toroidal transformer, a rectifier filter board, a motor driver, a stepper motor, and a square proximity switch. As the host computer, the Raspberry Pi captures camera images and identifies and locates the slow-growing mulberry cuttings. The robotic arm’s control board, acting as the lower computer, receives coordinate information and instructions from the Raspberry Pi, as well as position data from the proximity switch, to control the motor and facilitate the arm’s positioning and movement. The control board is also connected to the emergency stop switch and manipulator switch. In the case of any abnormality or fault, these switches allow the control board to take emergency action to protect the robotic arm’s safety. The manipulator switch starts and stops the robotic arm, while the emergency stop switch provides emergency protection. The power filter suppresses electromagnetic interference in the power supply, ensuring a stable and clean power signal for the system’s electronic components. The air switch provides additional safety and equipment protection. Toroidal transformers convert the incoming alternating current (AC) to the required voltage and current, minimizing magnetic flux leakage, electromagnetic radiation, noise, and vibration, thus ensuring smooth circuit operation. The rectifier filter board converts the AC into a direct current (DC) while reducing medium- and high-frequency noise to improve the circuit’s performance and stability. The motor driver receives instructions from the robotic arm control board and adjusts parameters such as the current, voltage, and frequency to control the motor’s speed and rotation angle. The stepper motor receives direction and pulse signals from the motor driver to determine the rotation direction and the angle of its shaft, thereby moving the nozzle on the mechanical arm’s end effector to the specified position.

This control system uses a Raspberry Pi 4B as the main controller, paired with a Raspberry Pi display. The lower controller is an STM32 microcontroller. The manipulator switch and emergency stop switch are both from the YJ139-LA38 series. The power filter is the CANNYWELL CW4L2-20A-T, and the air switch is a CHNT NXB-63 C32. We chose a toroidal transformer to step down the input voltage to the toroidal transformer from 220 V to 36 V. The power amplifier’s rectifier filter board supports an AC of 5-38V with a maximum current of 20 A, and it includes two 3300 μF 80 V capacitors. The motor driver is a Dingtuo DT5045 stepper motor driver. The motor is a 42-series stepper motor, with a 2-phase 2.0 A rating and a 50:1 gear ratio. The position sensor is an OMRON TL-Q5MC1-Z square proximity switch. The system for spraying foliar fertilizer uses a micro-peristaltic pump and a nozzle with a 0.5 mm pore size.

**Figure 6:**
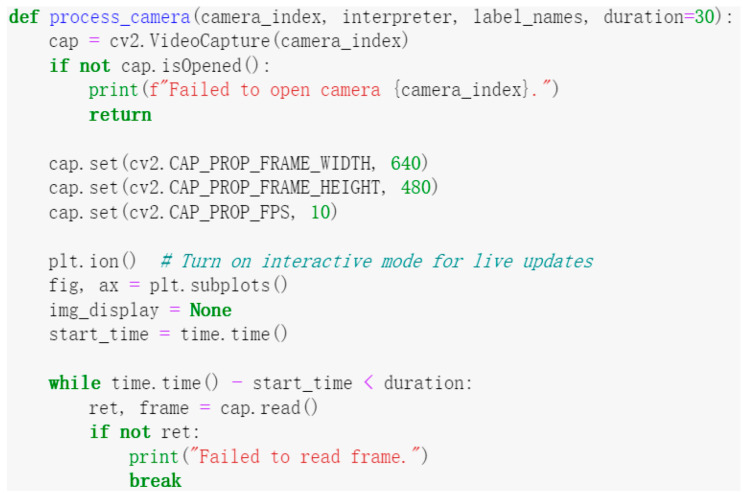
Camera control code.

**Figure 7:**
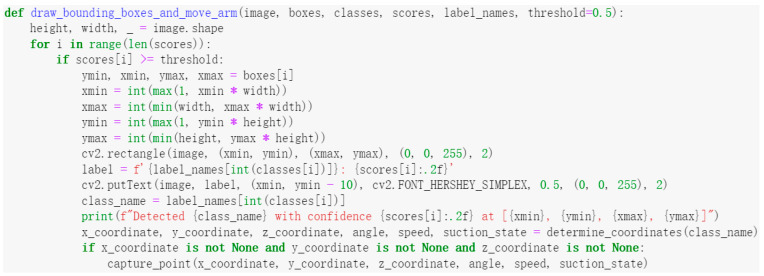
Bounding box drawing code.

After completing the hardware design and developing the control system, the next step was to write the control program code to implement the system’s functionality. Below is a portion of the code along with its corresponding function description. Figure 6 shows the code for controlling the camera, which results in a 30 s real-time video stream being captured from a specified camera and each frame being processed in real time. Figure 7 displays the code for drawing bounding boxes around mulberry cutting species in the camera image.

**Figure 8:**
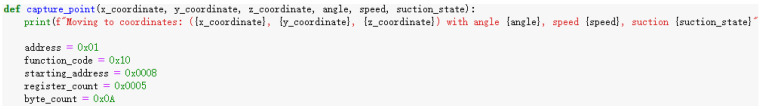
Robotic arm movement code.

Figure 7 presents the code for drawing bounding boxes around detected targets and guiding the robotic arm to specified coordinates based on the target category. Figure 8 shows the code for generating Modbus protocol commands to move the robotic arm to the specified coordinates and execute the adsorption action.

**Figure 9:**
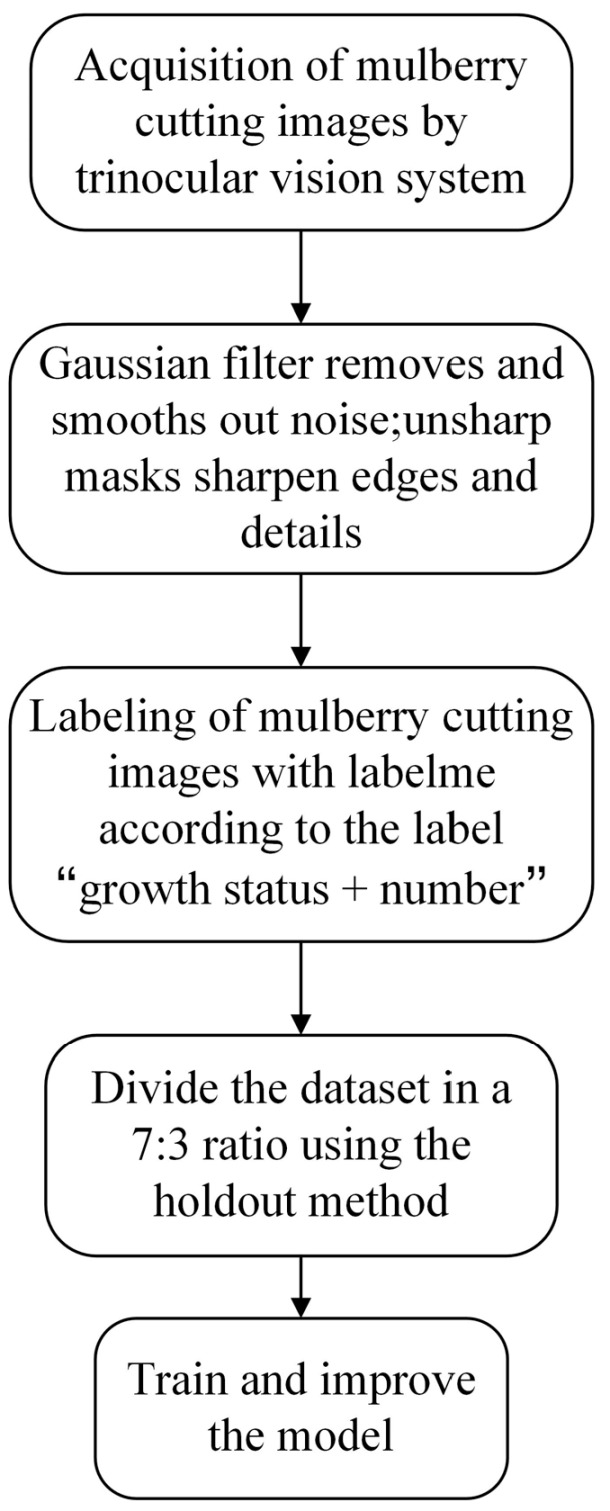
Flowchart of acquisition, processing, and calibration of images and training of models.

After building the system, the workflow needed to be carried out as shown in the Figure 9.

### 2.3. Datasets and Preprocessing

**Figure 10:**
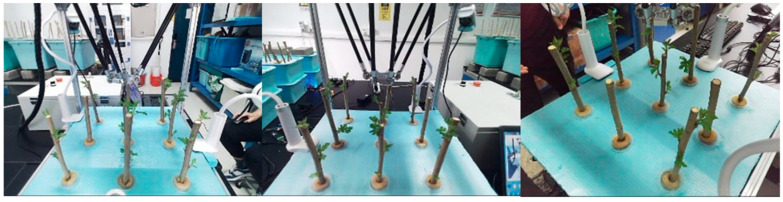
Samples of images captured by various cameras.

The experiment was conducted in the atomization laboratory in the LeiSi Building at Jiangsu University, with data collection occurring from 23 March to 14 April 2024. During this period, the relative humidity, temperature, and light intensity varied between 62% and 75%, 16 °C and 25 °C, and 411.7 Lux and 498.5 Lux, respectively. Images of the aeroponic mulberry tree cuttings, taken with different cameras, are presented in Figure 3. In order to capture the growth dynamics, three cameras were strategically placed: one in front of the mulberry plug, another on the left side, and a third 45° above and right behind. The cameras were autofocused with a resolution of 640 × 640 pixels, and all images were saved in a JPG format. Each image set included one photo from each of the three angles, with the arrangement of the cuttings adjusted between each capture. In total, 3000 images were collected throughout the study. Using a single camera angle often fails to fully capture the growth characteristics of mulberry cuttings. This limitation arises from factors such as the viewing angle, height, and occlusions caused by cuttings in the foreground, which can obscure those at the back. Such constraints can negatively affect the performance of recognition models. To overcome this challenge, the system utilized a trinocular imaging and recognition setup. The images taken on 23 March, 2 April, and 14 April 2024 (Figure 10) clearly demonstrate the healthy growth of the mulberry cuttings, with growth dynamics evident across all three dates.

**Figure 11:**
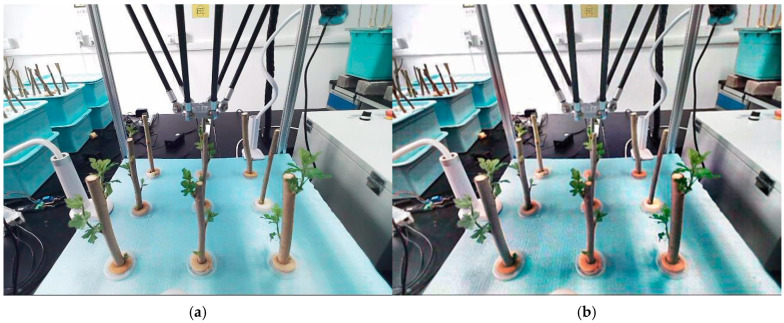
Comparison of before and after image processing. (**a**) Original image captured by the camera. (**b**) Pre-processed images.

To ensure high-quality model training and minimize potential errors, RGB images were preprocessed before data analysis. Gaussian filters were used to reduce and smooth out noise, while unsharp masks were applied to sharpen edges and details. Figure 11a shows the unprocessed image, and Figure 11b shows the processed image.

**Figure 12:**
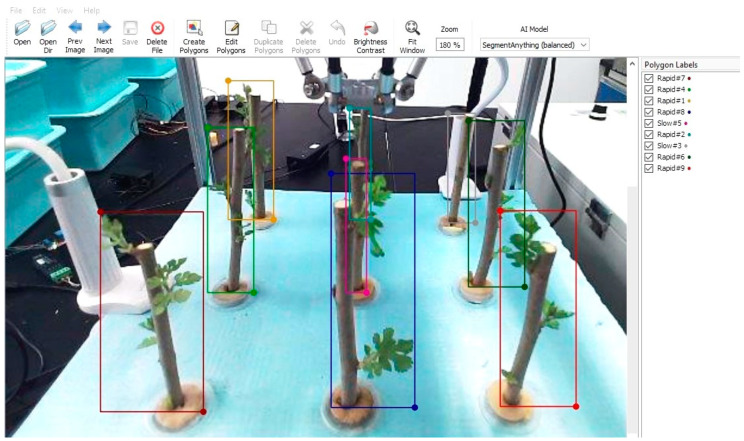
Image calibration.

The classification of slow and rapid growth was primarily based on the number of leaves, the total leaf area, and the frequency of dormancy periods being broken before growth resumed. Branches with larger characteristics were categorized as showing rapid growth, while others were classified as showing slow growth. In this study, Labelme, an open-source image annotation tool, was used to calibrate the captured mulberry tree cutting images. Normal-growing mulberry tree cuttings were labeled as “Rapid”, while slow-growing ones were labeled as “Slow”. The branches were labeled from #1 to #9, corresponding to their position relative to the robotic arm. The labeling tags combined the growth status and serial number of the mulberry cuttings in the format “growth status + serial number”, resulting in 18 label types: Rapid #1, Slow #1, Rapid #2, Slow #2, Rapid #3, Slow #3, Rapid #4, Slow #4, Rapid #5, Slow #5, Rapid #6, Slow #6, Rapid #7, Slow #7, Rapid #8, Slow #8, Rapid #9, and Slow #9. Figure 12 illustrates the process of picture calibration.

The holdout method was employed to split the dataset into a training set and a test set at a 7:3 ratio, enabling an accurate evaluation of the mulberry cutting recognition model’s performance. Specifically, 2100 images were allocated for training, while 900 images were reserved for testing.

### 2.4. YOLOv8n-Based Identification Model

YOLOv8n was selected as the baseline recognition model for this study. However, the original YOLOv8n model struggled with poor performance when applied to a dataset of collected mulberry cuttings. As a result, modifications were necessary to improve the model’s ability to accurately recognize mulberry cuttings.

To address this, several key improvements were made to the YOLOv8n model. First, we incorporated the Asymptotic Feature Pyramid Network (AFPN) progressive pyramid network, which was added after the backbone YOLOv8n Neck CSPDarknet [37]. This replaced the original FPN or PANet structure. The AFPN’s asymptotic feature fusion strategy allowed YOLOv8n to better combine feature information from different levels, significantly enhancing object detection precision.

In addition, the MS Block module was incorporated into the YOLOv8n C2f module [38,39]. This change involved replacing the original C2f module with a new network structure that included the MSBlock module. This modification boosted the model’s ability to extract multi-scale features, which, combined with new network parameter configurations, led to further improvements in both performance and detection precision.

DynamicATSS was incorporated into the YOLOv8n model to dynamically optimize key parameters based on the target scale, category, and dataset characteristics, further improving the performance and robustness of the object detection model [40].

#### 2.4.1. Parameter Settings

The training process for both the original YOLOv8n model and the improved YOLOv8n model was conducted using PyCharm (version 2022.1.4) with Python 3.9.7. The computer’s processor was an AMD R7-6800H, the graphics card was an RTX 3060, and the operating system was Windows 11. A total of 3000 images were collected using a trinocular vision system, with 1000 images from each camera. A total of 1000 images were selected from one camera as monocular samples, 500 from two cameras as binocular samples, and 1000 from all three cameras as trinocular samples. These samples were used to train YOLOv8n. Table 1 shows the hyperparameters of YOLOv8n when training on images acquired with different mesh sizes.

#### 2.4.2. Evaluation Metrics for YOLOv8n

The performance was evaluated using four indicators: the precision (Pr), recall rate (Re), F1 score (F1), and mean average precision (mAP). The calculation formulas are shown in Equations (1)–(4).

(1) $$Pr=\frac{TP}{TP+FP}$$

(2) $$Re=\frac{TP}{TP+FN}$$

(3) $$F1=2\times \frac{Pr\times Re}{Pr+Re}\;$$

(4) $$mAP=\int_{0}^{1}Pr\left(dRe\right)\;$$

where TP and FP denote true positive and false positive samples, respectively, and FN is a false negative sample.

### 2.5. Location of Mulberry Cuttings

#### 2.5.1. System Workflow

**Figure 13:**
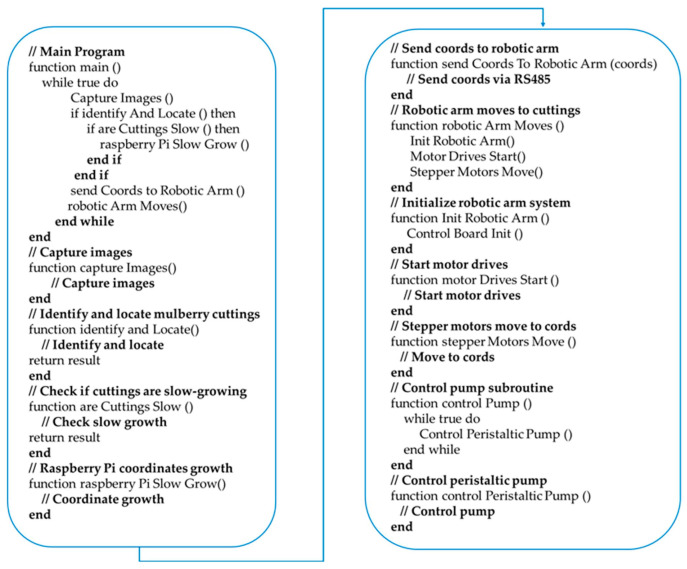
Pseudocode for automated mulberry tree cutting identification and fertilization system workflow.

The control system of the robotic arm is based on a Raspberry Pi as the upper computer and the robotic arm control board as the lower computer, with communication between the two via RS485. The Raspberry Pi 4B handles the identification and location of slow-growing mulberry cuttings in an aeroponic system and transmits the coordinate information and control instructions to the lower computer via RS485 communication (Appendix A). The lower computer then controls 42 stepper motors using a DT5045 motor drive to move the robotic arm above the slow-growing mulberry cuttings. Finally, the Raspberry Pi activates a vacuum pump to spray a solution promoting the growth of the cuttings. The pseudocode for the entire system’s operation is shown in Figure 13.

#### 2.5.2. Planting Board Placement and Hole Arrangement

The planting board used a 42 × 42 cm fireproof, heat-insulating foam board. The planting plate surface was a 3 × 3 matrix with 9 uniformly distributed planting holes and a single planting hole diameter of 40 mm; the center of the central planting holes and the geometric center of the planting plate completely coincided with the center of the space between any two adjacent planting holes for the 12 cm × 12 cm square grid layout. The center of the central hole aligned with the robotic arm’s ring, and the board’s four corners aligned with the robotic arm frame.

#### 2.5.3. Mulberry Cuttings Marked with Serial Numbers

**Figure 14:**
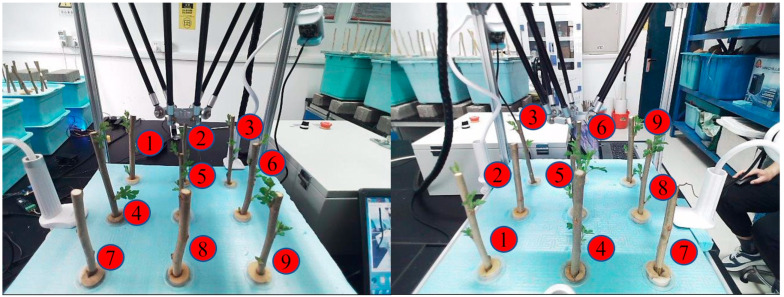
Schematic diagram showing mulberry cuttings with serial numbers.

The mulberry cuttings in the collected images were labeled with serial numbers. The serial numbers of mulberry cuttings in the same position in the three images within a group needed to be consistent. A schematic diagram of the labeling system is shown in Figure 14.

#### 2.5.4. Coordinate Conversion

After moving the robotic arm to a specific position using the robotic arm control software, an image was captured by each of the three cameras arranged above it, forming a group of images. The position of the robotic arm was then adjusted, and the process was repeated. In total, 845 groups of images were collected. The X, Y, and Z coordinates of the robotic arm’s position were determined using the software, as shown in Table 2. The target position coordinates were obtained by permuting and combining the X, Y, and Z coordinates.

The image annotation tool LabelMe was employed to calibrate the center point of the robotic arm’s end ring in the acquired position image. The X and Y coordinate values (image coordinates) in the JSON file generated after calibration corresponded to the X, Y, and Z coordinate values (real-world coordinates) of the robotic arm, as determined by the robotic arm testing software. These values were subsequently imported into an Excel table.

**Figure 15:**
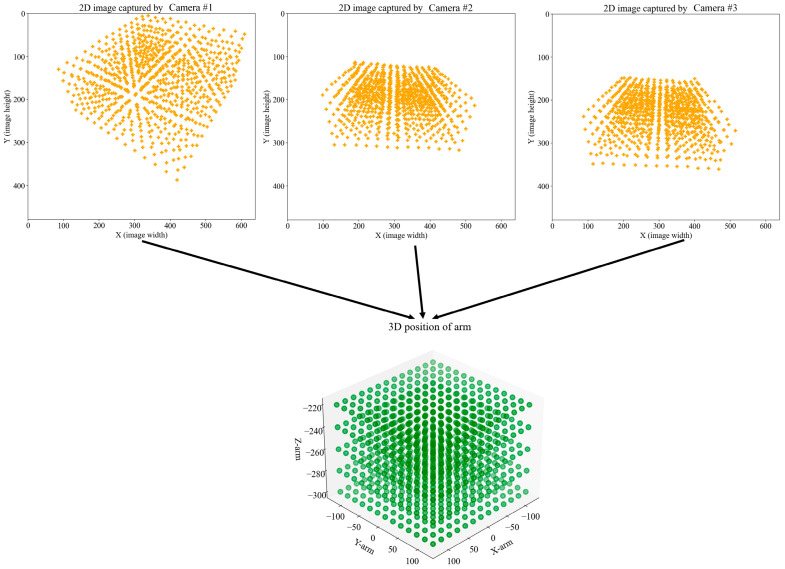
Scatter plot of the image coordinates captured by the camera versus the actual coordinates of the corresponding robotic arm.

After calibration with cameras 1, 2, and 3, the X and Y coordinates (image coordinates) in the generated JSON file were labeled as X_1_, Y_1_, X_2_, Y_2_, X_3_, and Y_3_, respectively. Python was then utilized to plot these coordinates (X_1_, Y_1_, X_2_, Y_2_, X_3_, Y_3_), along with the corresponding X, Y, and Z values, in scatter plots. These scatter plots are depicted in Figure 15.

### 2.6. Artificial Neural Network

**Figure 16:**
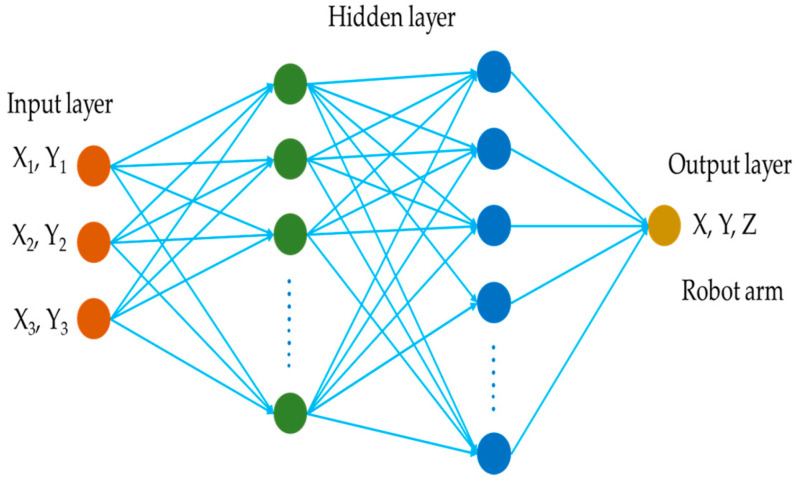
Structural diagram of the neural network model for predicting the coordinates of the robotic arm.

In the domain of robotic arm coordinate prediction, the Backpropagation Neural Network (BPNN) has emerged as a powerful computational tool. This model boasts a meticulously crafted architecture, comprising three distinct layers. The first layer, known as the input layer, serves as the primary data entry point. It processes the 2D coordinates of objects detected in images captured by multiple cameras, specifically X_1_, Y_1_, X_2_, Y_2_, X_3_, and Y_3_. This layer sets the stage for further data processing. The second layer, the hidden layer, acts as a bridge between the input and output layers. It plays a pivotal role in transforming the input data into a format that can be effectively utilized by the final layer. The output layer is where the predictions are generated. This layer took the processed data from the hidden layer and accurately determined the 3D positions of the robotic arm in motion, represented by the X, Y, and Z coordinates. The neural network model’s intricate structure, which precisely calculated the predicted coordinates of the robotic arm, is visually depicted in Figure 16. The model’s training process was equally sophisticated. It was configured to run for up to 500 iterations, utilizing the L-BFGS algorithm for weight optimization. To fine-tune the model’s performance, a rigorous hyperparameter tuning process was employed, leveraging 10-fold cross-validation. The inputs, X and Y coordinates extracted from images, were fed into the model. These inputs were processed using a variety of activation functions, including identity, logistic, tanh, and ReLU functions, to enhance the model’s learning capabilities. The number of neurons in the two hidden layers, denoted as nr1 and nr2, was systematically varied from 1 to 20. For each unique combination of parameters, the model underwent training, and the root mean square error (RMSE) was meticulously computed. The optimal configuration was identified by selecting the parameter set that yielded the lowest RMSE. Once the optimal configuration was determined, the BPNN model was retrained using the entire dataset. This comprehensive retraining ensured that the model was well equipped to handle unseen data, thereby ensuring its generalization performance. This step was crucial in ensuring that the model could reliably predict the robotic arm’s coordinates in real-world scenarios beyond the confines of the training data.

To streamline neural network training, feature scaling was applied. Normalization adjusted individual attributes (A) to account for variations in magnitude. Attribute normalization (A_norm_) was performed by subtracting the minimum value (A_min_) and dividing by the range between the maximum (A_max_) and minimum values, as demonstrated by the equation below:

(5) $$A_{norm}=\frac{A-A_{min}}{A_{max}-A_{min}}$$

Neural network training encompassed hyperparameter estimation and strategic feature selection. The process began with random multi-feature data fusion, followed by the exclusion of less significant features and prioritization of the most impactful ones. The efficacy of different configurations was evaluated by analyzing ANN outputs to identify the best variants, measured by the lowest root mean square error for cross-validation (RMSECV).

## 3. Results

**Figure 17:**
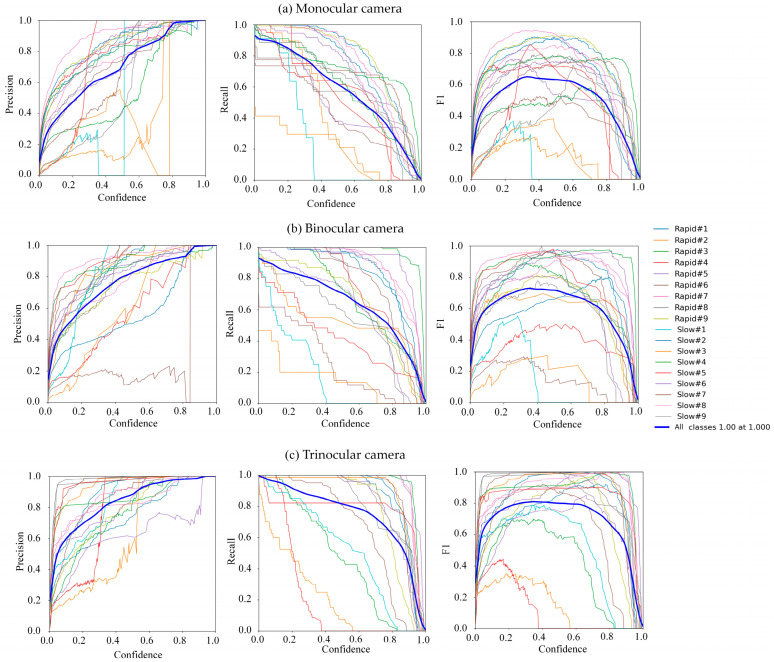
Comparative analysis of YOLOv8n model performance metrics across three distinct camera configurations: (**a**) monocular camera, (**b**) binocular camera, and (**c**) trinocular camera.

The recognition metrics—Pr, Re, F1, and mAP—were utilized as performance indicators to assess the growth state of mulberry cuttings. These metrics were deployed to assess the effectiveness of the YOLOv8n object detection model across various datasets derived from images captured by monocular, binocular, and trinocular cameras. Figure 17 illustrates the recognition performance of the YOLOv8n model for images collected using monocular, binocular, and trinocular cameras. Table 3 shows the precision, recall, and F1 score of YOLOv8n image recognition at different mesh counts. Table 4 displays the mean average precision for YOLOv8n image recognition at different mesh counts. The performance of the trinocular vision system was superior to that of the monocular and binocular vision systems. The precision of the trinocular vision was 15.51% and 8.96% higher than that of the monocular and binocular vision, respectively, and the mean average precision was 14.61% and 7.67% higher, respectively. The average precision, recall, and mean average precision of trinocular recognition for the current dataset were 68.99%, 67.52%, and 68.21%, respectively. Additionally, the F1 score of trinocular recognition was 0.68, calculated using Equation (4). For binocular recognition, the F1 scores were 60.03%, 60.55%, 60.54%, and 0.60, respectively. In comparison, the Pr, Re, mAP, and F1 scores for monocular recognition were 53.47%, 55.21%, 53.60%, and 0.54, respectively. The trinocular recognition system was more comprehensive in capturing the shape, size, and surface details of and other information on mulberry cuttings. In contrast, the monocular and binocular recognition systems may not have been able to obtain all the key information on mulberry cuttings due to limitations in viewing angles. Consequently, the trinocular recognition system performed better. Moreover, by comparing parameters such as the Pr, Re, mAP, and F1 score, it is evident that the image recognition performance of the trinocular system surpassed that of the monocular and binocular systems. Additionally, the precision of the recognition model generally increased with the size of the training set, indicating that the image acquisition effectiveness of the trinocular system was superior.

**Figure 18:**
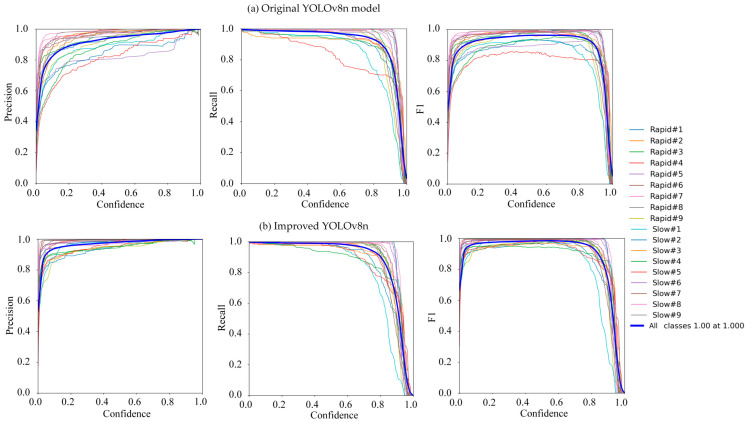
Evaluation of Yolov8n model’s performance metrics pre- and post-enhancement.

**Figure 19:**
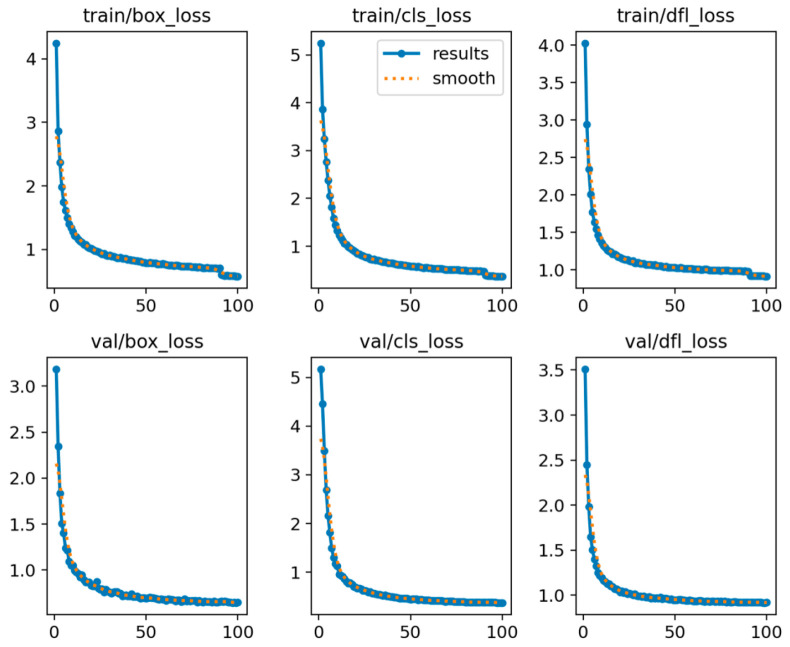
Loss profile plots for the training and validation sets for the YOLOv8-improve model.

Below are the performance evaluation plots and tables showing the results obtained after training YOLOv8n, YOLOv10, Faster RCNN, and the improved YOLOv8n (YOLOv8-improve) using the mulberry cutting dataset. Figure 18 shows a performance comparison between the original YOLOv8 model and the YOLOv8-improve model. Figure 19 shows the loss profile of the YOLOv8-improve model for the training and validation sets.

Combining the results shown in Figure 18 and Figure 19, although the mulberry cutting dataset used in this paper (3000 images) was small, the recall and loss were normal, and there was no overfitting.

Table 5 shows the precision, recall, and F1 score data for different models, and Table 6 shows the mean average precision for different models.

This study appraised the performance (Pr, Re, F1, and mAP) of various detection models, including the original YOLOv8n (Figure 18), improved YOLOv8n (Figure 18), YOLOv10, and Faster R-CNN, on the mulberry cutting dataset. The original YOLOv8n model achieved recognition Pr, Re, mAP, and F1 scores of 86.08%, 86.83%, 88.44%, and 0.8645, respectively. In contrast, the improved YOLOv8n model achieved recognition Pr, Re, mAP, and F1 scores of 93.11%, 93.40%, 94.48%, and 0.93%, respectively. The Progressive Feature Pyramid Network (AFPN) was introduced to replace the original FPN or PANet structure of YOLOv8n, reducing the semantic gap between different hierarchical features and enhancing the model’s detection performance for small targets. Additionally, the MSBlock module was incorporated into the C2f module, improving the size and structure of the convolutional kernels and optimizing the feature fusion method. This enhancement boosted the model’s performance when processing multi-scale information. Furthermore, the CIoU loss function of YOLOv8n was replaced with Focal_XIoU to address the imbalance between positive and negative samples and to improve the precision of bounding box regression. The DynamicATSS module was also incorporated into the label assignment strategy, enhancing the model’s detection and generalization capabilities while reducing the discrepancy between classification and IoU scores. By comparing the recognition precision, recall, mean average precision, and F1 score, it is evident that the improved YOLOv8n model significantly outperformed the original YOLOv8n model in recognizing the acquired images.

As illustrated in Table 5, the optimized YOLOv8n model exhibited a notable improvement in both Pr (93.11%) and Re (93.40%) over the original YOLOv8n, which had values of 86.08% and 86.83%, respectively. This indicates a substantial enhancement in its overall detection capabilities. Additionally, YOLOv8n outperformed YOLOv10 in terms of recall, although the latter showed a marginally better precision score. This trade-off between precision and recall suggests that the model’s suitability can vary depending on whether minimizing false positives or maximizing true detections is more critical for the application. On the other hand, Faster R-CNN performed less effectively with a Pr at 55.10% and an Re at 75.50%, signaling that it might not be optimal for the current task and could benefit from further refinement. These findings highlight the importance of carefully choosing models based on specific detection objectives and the balance between precision and recall. Additionally, Table 6 outlines the mAP values for each model for the mulberry cutting dataset. The value of the mAP for YOLOv8n was 88.43%, while YOLOv8n-improve achieved a higher average of 94.48%, indicating better overall detection performance for the improved model. Our results, based on advanced YOLOv8n, outperform those of Wang et al. [41], who utilized a spatial channel decoupled downsampling approach. By first enhancing the channels with pointwise convolution (PW) and then reducing the resolution through depth-wise convolution (DW) in the YOLOv10-S framework, they achieved a 0.7% improvement in the average precision by minimizing information loss.

The R-squared (R^2^) value, mean squared error (MSE), and root mean squared error (RMSE) were used to evaluate the differences between the model’s predicted values and the true values. These metrics for the X, Y, and Z coordinates are presented in Table 7. The model’s test performance for predicting the robotic arm’s coordinates was as follows: For the X coordinate, the test set achieved an R^2^ of 99.90% and an RMSE of 0.006. For the Y coordinate, the test set had an R^2^ of 99.90% and an RMSE of 0.006. For the Z coordinate, the test set reached an R^2^ of 99.90% and an RMSE of 0.012. The analysis of these metrics indicated that the error between the predicted and actual coordinates was very small and nearly negligible. Therefore, the coordinate conversion for slow-growing mulberry cuttings was highly accurate, enabling the precise location of the cuttings.

**Figure 20:**
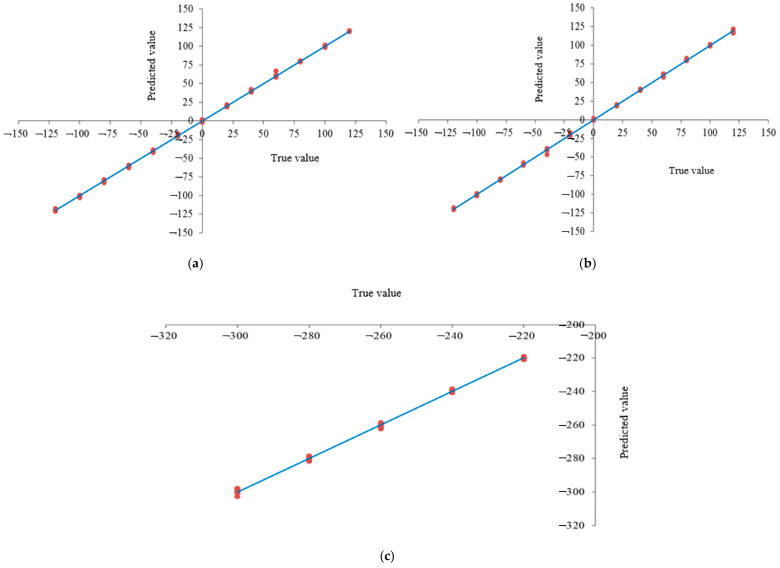
Scatter plots of the predicted and true values for the robotic arm’s coordinates: (**a**) X-arm, (**b**) Y-arm, and (**c**) Z-arm.

The scatter plot (Figure 20) depicting a comparison between the actual and predicted X, Y, and Z coordinates of the robotic arm offers valuable insights into the system’s positional accuracy. By comparing the model’s predicted positions against the true values obtained from the manipulator’s sensors, we can evaluate the precision of the arm’s movements along all three axes. A closer alignment between the predicted and actual coordinates indicates higher accuracy in the manipulator’s movements, which directly impacts the spraying precision. When the predicted coordinates closely match the true coordinates, it suggests that the spraying mechanism will operate with greater accuracy, leading to better coverage and the more precise targeting of the intended areas. Thus, the correlation and any discrepancies in the scatter plot are critical for understanding the spraying accuracy and identifying potential areas where positional errors could affect the robotic arm’s performance in spraying tasks.

The average precision, recall, and mean average precision of trinocular recognition in the current dataset were 68.99%, 67.52%, and 68.21%, respectively. Additionally, the F1 score of trinocular recognition was 0.68, calculated using Equation (4). For binocular recognition, the Pr, Re, mAP, and F1 scores were 60.03%, 60.55%, 60.54%, and 0.60, respectively. In comparison, the Pr, Re, mAP, and F1 scores for monocular recognition were 53.47%, 55.21%, 53.60%, and 0.54, respectively. The trinocular recognition system was more comprehensive in capturing the shape, size and surface details of and other information on mulberry cuttings. In contrast, the monocular and binocular recognition systems may not have been able to obtain all the key information on mulberry cuttings due to limitations in viewing angles. Consequently, the trinocular recognition system performed better. Moreover, by comparing parameters such as the Pr, Re, mAP, and F1 score, it is evident that the image recognition performance of the trinocular system surpassed that of the monocular and binocular systems. Additionally, the precision of the recognition model generally increased with the size of the training set, indicating that the image acquisition effectiveness of the trinocular system was superior.

The original YOLOv8n model achieved recognition Pr, Re, mAP, and F1 scores of 86.08%, 86.83%, 88.44%, and 0.8645, respectively. In contrast, the improved YOLOv8n model achieved recognition Pr, Re, mAP, and F1 scores of 93.11%, 93.40%, 94.48%, and 0.93, respectively. The Progressive Feature Pyramid Network (AFPN) was introduced to replace the original FPN or PANet structure of YOLOv8n, reducing the semantic gap between different hierarchical features and enhancing the model’s detection performance for small targets. Additionally, the MSBlock module was incorporated into the C2f module, improving the size and structure of the convolutional kernels and optimizing the feature fusion method. This enhancement boosted the model’s performance when processing multi-scale information. Furthermore, the CIoU loss function of YOLOv8n was replaced with Focal_XIoU to address the imbalance between positive and negative samples and to improve the precision of bounding box regression. The DynamicATSS module was also incorporated into the label assignment strategy, enhancing the model’s detection and generalization capabilities while reducing the discrepancy between classification and IoU scores. By comparing the recognition precision, recall, mean average precision, and F1 score, it is evident that the improved YOLOv8n model significantly outperformed the original YOLOv8n model in recognizing the acquired images.

The analysis of the R^2^ value, mean square error, root mean square error, and other parameters derived from the predicted coordinates of the robotic arm using the trained neural network model indicated that the error between the predicted and actual coordinates was very small and nearly negligible. Therefore, the coordinate conversion for slow-growing mulberry cuttings was highly accurate, enabling the precise location of the cuttings.

## 4. Discussion

Liyang [42] designed an intelligent control system for water and fertilizer integration in a tomato greenhouse. Compared with that, the intelligent spraying system designed in this paper is able to spray foliar fertilizer more accurately after target detection and localization, reducing the waste of fertilizer and lowering the cost. The improved YOLOv8n model used in this paper is more efficient compared to S Li’s model [43] in intelligent decision-making and control regarding water and fertilizer that integrate multiple sources of data inputs. Moreover, the intelligent spraying system designed in this paper is less affected by environmental factors. Kim et al. [44] presented an intelligent spraying system for the semantic segmentation of fruit trees in pear orchards. The system was trained with images categorized into five distinct classes. The trained deep learning model achieved a precision of 83.79%. Compared to that system, the initial precision of the target detection model for the intelligent spraying system designed in this paper was 88.43%, and the precision of the improved target detection model reached 94.48%, which was a significant improvement in precision. In summary, the intelligent spraying system designed in this paper has certain advantages and competitiveness, but at the same time, it has certain shortcomings. The advantages of this study include the use of target detection technology to accurately identify slow-growing crop plants and an intelligent control system for precise and quantitative fertilization at specific locations. This approach reduces fertilizer waste and lowers production costs. Additionally, despite limited resources (the fixed arithmetic power of a Raspberry Pi), the recognition model achieves a balance between speed and precision, thereby reducing costs.

(1) Regarding the spraying of foliar fertilizer for 20 s after the model has quickly detected slow-growing mulberry cuttings, 20 s is just our own setting, and the optimal foliar fertilizer spraying duration needs to be investigated experimentally to improve the growth rate of mulberry cuttings while reducing the consumption of foliar fertilizer.
(2) The current 1 h spraying interval for foliar fertilizer is only suitable for the current growth state of the mulberry cuttings; with the gradual growth of the mulberry cuttings, the foliar fertilizer they need will increase, and continuing to spray it according to the current spraying interval and spraying time will lead to insufficient nutrients. Several experiments are needed to optimize the spraying interval and duration.
(3) It is necessary to adjust the angle and height of the camera according to the growth of mulberry tree cuttings in order to better detect the growth of mulberry tree cuttings.

In our experimental study, we observed that 25% of the mulberry cuttings exhibited slower growth rates. By employing our intelligent spraying system to target and fertilize only these underperforming cuttings, we achieved a notable reduction in the overall fertilizer usage. This targeted approach to spraying, as opposed to the conventional uniform application method, significantly decreased fertilizer consumption. The potential cost savings and environmental benefits of this targeted strategy warrant further investigation in future research endeavors. Beyond reducing material costs, our targeted fertilization method aligns with eco-friendly agricultural practices by minimizing waste and conserving valuable resources.

In the future, we will test the system in a greenhouse or in an external environment with the following research focuses:

(1) The environmental adaptability of the system.
(2) The effectiveness of the system in saving fertilizer.
(3) The actual identification and localization capabilities of the system.
(4) Improvements to the model made according to the actual situation.

Additionally, we recognize the critical role of sustainable energy solutions in enhancing the efficiency and autonomy of agricultural monitoring systems. As emphasized by Abidin et al. [45], optimizing energy harvesting for low-power sensors in wireless sensor networks is essential for the long-term sustainability of such systems. By integrating the sustainable energy solutions proposed in their research, we can further improve the efficiency and reduce the environmental impact of our intelligent spraying system.

Since our trinocular vision system has some technical limitations, in order to further improve the system performance, we will draw on the advanced 3D imaging technology solutions proposed by Hu K [46], Li X [47], etc., and continue to optimize the system architecture by leveraging the capabilities of cutting-edge technology.

Looking ahead, we aim to refine the system’s limitations and minimize environmental influences to enhance its contribution to precision and smart agriculture. While the framework has been assessed in controlled settings, subsequent research should focus on deploying it in semi-controlled greenhouse environments over multiple growing seasons. This approach would offer valuable insights to optimize its adaptability and performance in a wider range of real-world scenarios.

## 5. Conclusions

In this study, we compared the performance of monocular, binocular, and trinocular recognition systems for monitoring the growth of mulberry tree plugs. The trinocular system outperformed the others, achieving an average recognition precision of 68.21%, which was 14.61% and 7.67% higher than that of the monocular and binocular systems, respectively. By comparing YOLOv8n and YOLOv10, the simulation-validated YOLOv8n model achieved an average recognition precision of 94.48%, which was 6.05% higher than that of the original model. This improvement is attributed to the integration of the Asymptotic Pyramid Network (AFPN), the MSBlock module, the Dynamic Allocation Strategy (DynamicATSS), and the XIoU loss function. Additionally, the image–mechanical arm coordinate prediction model, based on a neural network, achieved a maximum positioning error of ≤1.3%. The parallel robotic arm foliar fertilizer spraying system, supported by the use of this model, can accurately perform the intelligent identification and localization of and fertilizer application for weak seedlings in an aerosol cultivation environment. In the future, we aim to further enhance the precision of the target detection model, improve the localization accuracy, minimize the waste of the mulberry foliar fertilizer solution, and contribute to the development of precision and smart agriculture.

## Figures and Tables

**Table 1:** Hyperparameters of YOLOv8n for training.

Parameter	Value
Split ratio	0.3
Batch size	4
Learning rate	0.0001
Epoch	100
Image size	(640, 640)

**Table 2:** Mapping the robotic arm’s path in 3D space.

X-Arm	Y-Arm	Z-Arm
0	0	−220
20	20	−240
40	40	−260
60	60	−280
80	80	−300
100	100	-
120	120	-
−20	−20	-
−40	−40	-
−60	−60	-
−80	−80	-
−100	−100	-
−120	−120	-

**Table 3:** Precision, recall, and F1 score of YOLOv8n for image recognition with different mesh numbers.

Mesh Number	Evaluation Index		
	Precision (%)	Recall (%)	FI Score
Monocular	53.48	55.21	36.00
Binocular	60.03	60.55	60.00
Trinocular	68.99	67.52	68.00

**Table 4:** Mean average precision of YOLOv8n for image recognition with different mesh numbers.

Mesh Number	Mean Average Precision (%)		
	Minimum	Average Value	Maximum
Monocular	0	53.60	72.55
Binocular	0	60.54	81.35
Trinocular	0	68.21	89.60

**Table 5:** Precision, recall, and F1 score of various models.

Detection Model	Evaluation Metrics		
	Precision (%)	Recall (%)	F1 Score
YOLOv8n	86.08	86.83	87.00
YOLOv8n-AFPN	91.33	90.66	91.00
YOLOv8n-MSBlock	87.44	87.11	87.00
YOLOv8n-XIoU	91.27	93.38	92.00
YOLOv8n-DynamicATSS	92.32	93.29	92.80
YOLOv8n-improve	93.11	93.40	93.00
YOLOv10	89.10	85.60	86.00
Faster RCNN	55.10	75.50	60.00

**Table 6:** Mean average precision of various models.

Detection Model	Mean Average Precision (%)		
	Minimum	Average Value	Maximum
YOLOv8n	0.08	88.43	98.69
YOLOv8n-AFPN	0.30	92.13	99.10
YOLOv8n-MSBlock	0.04	89.57	99.01
YOLOv8n-XIoU	0.34	93.47	99.24
YOLOv8n-DynamicATSS	0.01	93.83	99.06
YOLOv8n-improve	0.40	94.48	99.27
YOLOv10	0.06	84.90	98.54
Faster RCNN	0.02	58.70	65.92

**Table 7:** Evaluation metrics of the artificial neural network for the calculation of the arm coordinates.

Arm Coordinates	Optimal Parameters	Training			CV			Test		
		R^2^	RMSE	MSE	R^2^	RMSE	MSE	R^2^	RMSE	MSE
X	(6, 3, tanh)	99.90	0.005	0.00003	99.70	0.009	0.00008	99.90	0.006	0.00004
Y	(6, 5, tanh)	99.90	0.006	0.00004	99.80	0.008	0.00006	99.90	0.006	0.00004
Z	(10, 5, tanh)	99.80	0.013	0.00169	0.998	0.013	0.00169	0.999	0.012	0.00144

## Data Availability

The data will be made available on request.

## Appendix A

def detect_objects(image, interpreter, label_names):

  input_details = interpreter.get_input_details()

  output_details = interpreter.get_output_details()

  input_data = preprocess_image(image)

  interpreter.set_tensor(input_details[0][‘index’], input_data)

  interpreter.invoke()

  scores = interpreter.get_tensor(output_details[0][‘index’])[0]

  boxes = interpreter.get_tensor(output_details[1][‘index’])[0]

  classes = interpreter.get_tensor(output_details[3][‘index’])[0]

  return boxes, classes, scores

def determine_coordinates(class_name):

  coordinates = {

    "Slow#1": (−900, 900, 5),

    "Slow#2": (0, 900, 5),

    "Slow#3": (900, 900, 5),

    "Slow#4": (−900, 0, 5),

    "Slow#5": (0, 0, 5),

    "Slow#6": (900, 0, 5),

    "Slow#7": (−900, −900, 5),

    "Slow#8": (0, −900, 5),

    "Slow#9": (900, −900, 5)

  return coordinates.get(class_name, (None, None, None))}

def main():

  camera_indices = [0, 2, 4]

  interpreter = tflite.Interpreter(model_path = ”efficientdet.tflite”)

  interpreter.allocate_tensors()

  label_names = [line.rstrip(‘\n’) for line in open(“labelmap1.txt”)]

  label_names = np.array(label_names)

  # Serial port configuration

  port = ‘/dev/ttyUSB0’ # Replace with the actual serial port

  baudrate = 9600

  parity = serial.PARITY_EVEN

  stopbits = serial.STOPBITS_ONE

  bytesize = serial.EIGHTBITS

  timeout = 5 # Increase timeout for serial communication
